# Supplementary material for: Programmable high-dimensional Hamiltonian in a photonic waveguide array
Source: Nat Commun. 2024 Jan 2;15:50. doi: 10.1038/s41467-023-44185-z (PMC10761861; doi:10.1038/s41467-023-44185-z)
Supplement: Supplementary file 1 — Supplementary Information [file 41467_2023_44185_MOESM1_ESM.pdf]

Supplementary Information for
Programmable high-dimensional Hamiltonian in a photonic waveguide array

Yang Yang,[1] Robert J. Chapman,[1,2] Ben Haylock,[3,4] Francesco Lenzini,[3,5]
Yogesh N. Joglekar,[6] Mirko Lobino,[3,7,8] and Alberto Peruzzo[1,9]

[1] *Quantum Photonics Laboratory and Centre for Quantum Computation and Communication Technology, RMIT University, Melbourne, VIC 3000, Australia*
[2] *ETH Zurich, Optical Nanomaterial Group, Institute for Quantum Electronics, Department of Physics, 8093 Zurich, Switzerland*
[3] *Centre for Quantum Computation and Communication Technology (Australian Research Council), Centre for Quantum Dynamics, Griffith University, Brisbane, QLD 4111, Australia*
[4] *Institute for Photonics and Quantum Sciences, SUPA, Heriot-Watt University, Edinburgh EH14 4AS, United Kingdom*
[5] *Institute of Physics, University of Muenster, 48149 Muenster, Germany*
[6] *Department of Physics, Indiana University Purdue University Indianapolis (IUPUI), Indianapolis, Indiana 46202, USA*
[7] *Department of Industrial Engineering, University of Trento, via Sommarive 9, 38123 Povo, Trento, Italy*
[8] *INFN–TIFPA, Via Sommarive 14, I-38123 Povo, Trento, Italy*
[9] *Qubit Pharmaceuticals, Advanced Research Department, Paris, France*

SUPPLEMENTARY FIGURES

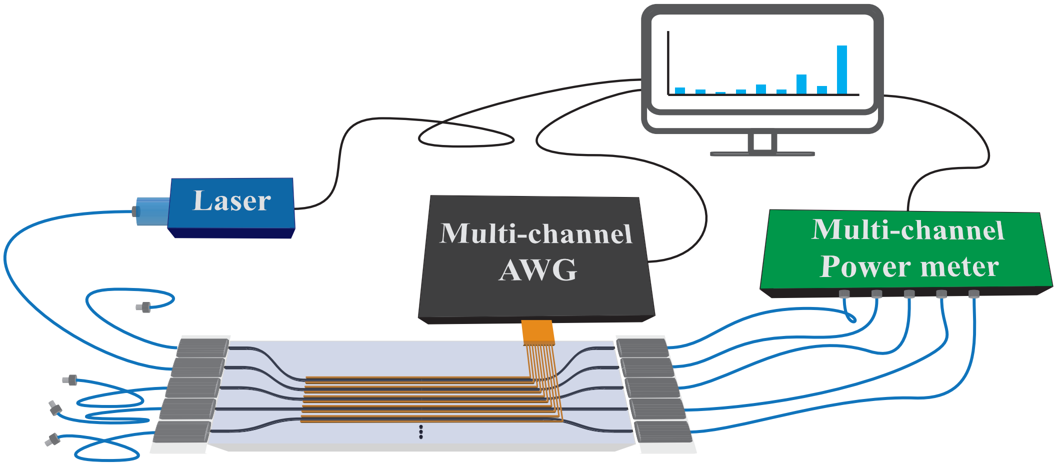

Supplementary Fig 1: Schematic of the setup. Two fiber arrays are coupled to the chip as input and output. An 808 nm polarized laser is connected to the input fiber array. Multi-channel arbitrary waveform generators are connected to the on-chip electrodes to generate control pulses. A multi-channel power meter is used to measure the chip output.

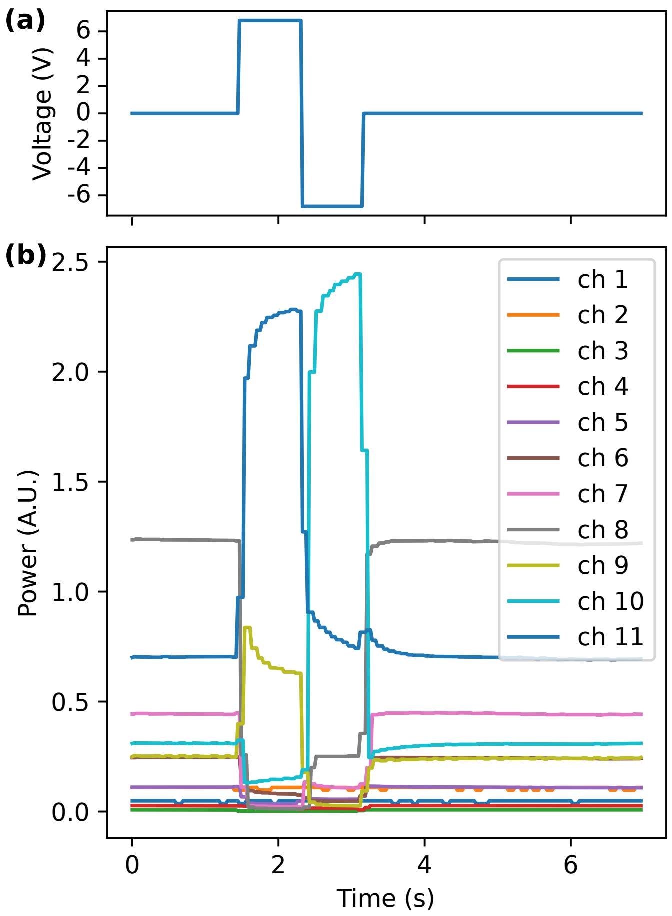

Supplementary Fig 2: Chip response example of SSH experiment (V=6.7 V) (a) The 1.66 s long 6.7 V non-biased square pulses from one of the AWG channels.(b) Output power measurement of SSH experiment with the pulsing shown in (a), following the pattern shown in Fig 3 (b).

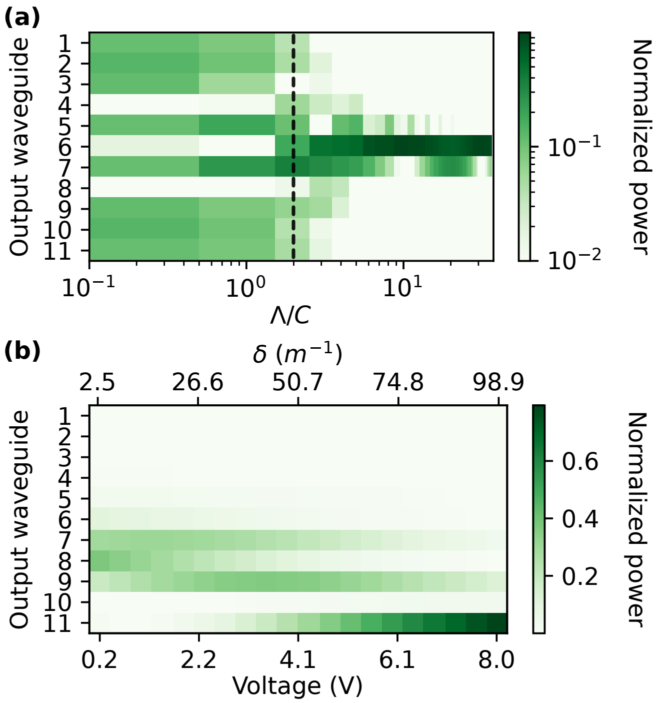

Supplementary Fig 3: Simulations of the voltage-controlled output distribution. (a) Simulation of implementation of Aubry-André model with the device. (b) Simulation of implementation of SSH model with the device.

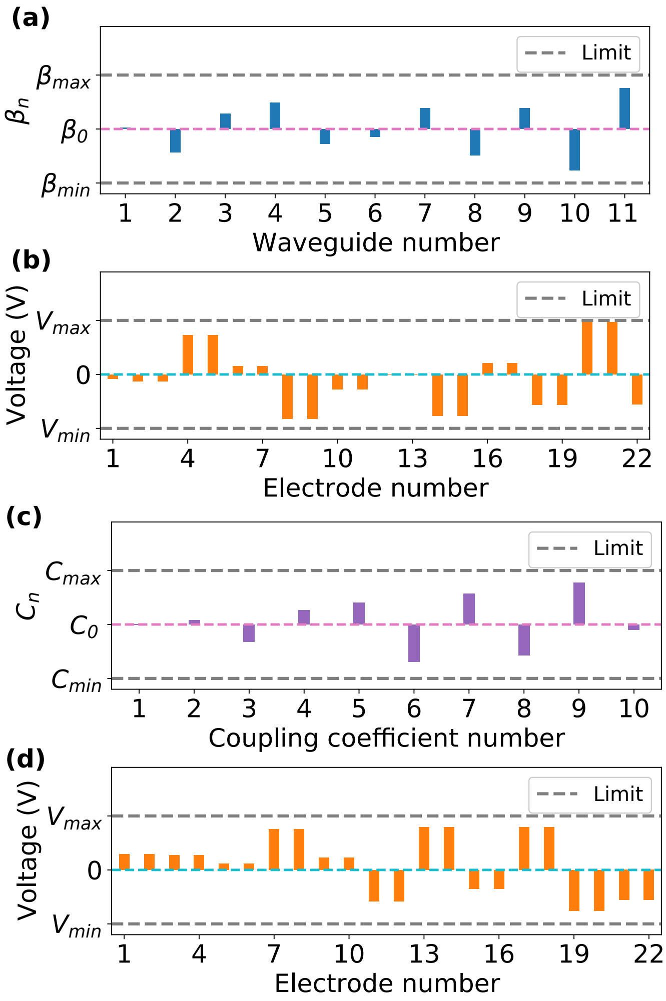

Supplementary Fig 4: Hamiltonian and corresponding voltage setting for Anderson localization experiments. (a) Propagation constants of each waveguide are randomly distributed within a range. (b) We paired every two electrodes between waveguides and applied pulses on all electrodes with random voltage amplitudes in a range. (c) Coupling constants between waveguides are randomly distributed within a range. (d) We paired every two electrodes on the top of each waveguide and applied pulses on all electrodes with random voltage amplitudes in a range.
